# Imaging Mass Spectrometry for the Classification of Melanoma Based on *BRAF*/*NRAS* Mutational Status

**DOI:** 10.3390/ijms24065110

**Published:** 2023-03-07

**Authors:** Rita Casadonte, Mark Kriegsmann, Katharina Kriegsmann, Helene Streit, Rolf Rüdiger Meliß, Cornelia S. L. Müller, Joerg Kriegsmann

**Affiliations:** 1 Proteopath GmbH, 54296 Trier, Germany; 2 Institute of Pathology, University Hospital Heidelberg, 69120 Heidelberg, Germany; 3 Institute of Pathology Wiesbaden, 69120 Heidelberg, Germany; 4 Department of Hematology Oncology and Rheumatology, University Hospital Heidelberg, 69120 Heidelberg, Germany; 5 Department of Medicine, Faculty of Medicine and Dentistry, Danube Private University, 3500 Krems, Austria; 6 Institute für Dermatopathologie, 30519 Hannover, Germany; 7 MVZ für Histologie, Zytologie und Molekulare Diagnostik Trier, 54296 Trier, Germany

**Keywords:** *BRAF*, classification, diagnostics: imaging mass spectrometry, MALDI, melanoma, *NRAS*

## Abstract

Mutations of the oncogenes v-raf murine sarcoma viral oncogene homolog B1 (*BRAF*) and neuroblastoma RAS viral oncogene homolog (*NRAS*) are the most frequent genetic alterations in melanoma and are mutually exclusive. *BRAF* V600 mutations are predictive for response to the two *BRAF* inhibitors vemurafenib and dabrafenib and the mitogen-activated protein kinase kinase (MEK) inhibitor trametinib. However, inter- and intra-tumoral heterogeneity and the development of acquired resistance to *BRAF* inhibitors have important clinical implications. Here, we investigated and compared the molecular profile of *BRAF* and *NRAS* mutated and wildtype melanoma patients’ tissue samples using imaging mass spectrometry-based proteomic technology, to identify specific molecular signatures associated with the respective tumors. SCiLSLab and R-statistical software were used to classify peptide profiles using linear discriminant analysis and support vector machine models optimized with two internal cross-validation methods (leave-one-out, k-fold). Classification models showed molecular differences between *BRAF* and *NRAS* mutated melanoma, and identification of both was possible with an accuracy of 87–89% and 76–79%, depending on the respective classification method applied. In addition, differential expression of some predictive proteins, such as histones or glyceraldehyde-3-phosphate-dehydrogenase, correlated with *BRAF* or *NRAS* mutation status. Overall, these findings provide a new molecular method to classify melanoma patients carrying *BRAF* and *NRAS* mutations and help provide a broader view of the molecular characteristics of these patients that may help understand the signaling pathways and interactions involving the altered genes.

## 1. Introduction

Malignant melanoma (MM) is an aggressive and deadly skin cancer with an increasing incidence among fair-skinned populations and in regions of lower latitude [1]. MM can arise on apparently healthy skin or from a pre-existing nevus. About 86% of melanomas are attributed to ultraviolet (UV) exposure [2]. Only 20 to 30 percent of melanomas are found in existing moles, while 70 to 80 percent arise on apparently normal skin [3]. Ultraviolet radiation represents a major risk factor for the development of cutaneous melanoma genesis through direct DNA damage. Overall survival at 5 years depends on histopathological features such as the thickness of the melanoma and clinicopathological staging including lymph node status and the presence of distant metastasis. Mutational testing is currently recommended to enable patients for treatment with targeted therapies. Several types of gene mutations are present in cutaneous melanoma and play a key role in the development and progression of the disease. Mutations of the oncogenes *BRAF* (v-raf murine sarcoma viral oncogene homolog B1) *NRAS* (neuroblastoma RAS viral oncogene homolog) and *KRAS* (Kirsten rat sarcoma viral oncogene) are among the most common genetic alterations in skin melanoma, leading to increased signaling activity of the mitogen-activated protein kinase (MAPK) pathway and thereby promoting tumor growth and disease progression [4,5,6]. *BRAF* and *NRAS* mutations have been identified in 50% and 8% of melanoma, respectively. More than 20 BRAF mutations have been described in melanoma. Among them, *BRAF* V600E mutation accounts for the majority (80–90%) of all *BRAF* mutations in melanoma [7,8]. More than 80% of *NRAS* mutated melanoma carry the substitution of glutamine with arginine, lysine, or leucine at position p.61 (*NRASQ61R/K/L*) [9]. *BRAF* V600 inhibitors have shown significant improvement in the prognosis and survival of patients with advanced disease [6,10]. Specifically, the Food and Drug Administration (FDA) has approved the two *BRAF* inhibitors vemurafenib [11] and dabrafenib [12], inducing confirmed responses in more than 50% of patients, and the MEK (mitogen-activated protein kinase kinase) inhibitor trametinib, which showed confirmed response rates in 20% of patients [13].

It has been known that *BRAF* V600 mutations are also frequently found in benign and dysplastic melanocytic nevi. This indicates that additional cellular changes are needed for melanocyte transformation, which may include inactivation of genes involved in DNA repair, activation of other protein kinases and signaling cascades, or loss of tumor suppressor [14,15].

Despite progress in the treatment of advanced melanoma, many questions still remain, and for the majority of melanoma patients, prognosis remains poor. It has been postulated that *BRAF* and *NRAS* mutations are most likely due to clonal heterogeneity within the tumor [16]. The involvement of powerful exogenous mutagens in the pathogenesis of melanoma, such as ultraviolet light, likely explains the elevated clonal mutational load in these cancers. Tumor heterogeneity in melanoma has been associated with drug sensitivity, immunotherapy resistance and dedifferentiation of melanoma cells [17,18,19]. The different clinical, histopathological, and molecular characteristics of patients affected by malignant melanoma are important for patient management and therapy stratification.

Our understanding of the molecular features in MM is still limited, and identification of novel biomarkers that define patient populations that benefit from specific treatments is needed. As the high mutational load in many melanomas results in a large variety of tumor antigens, the application of proteomic methods to investigate the proteomic landscape of melanoma is of particular interest. Imaging mass spectrometry (IMS) is a powerful technology capable of providing proteomics information directly from tissue sections, and it simultaneously reveals the spatial distribution of molecular signatures without the requirement for special labels [20]. The IMS analysis can be restricted to specific regions in the tissue where data of different cell types can be extracted for statistical characterization of proteome differences [21]. IMS-based studies have been performed to distinguish molecular signatures of metastatic melanoma tumors from lymph nodes free of tumor and to identify a set of proteins correlating with patient prognosis [22,23]. Using IMS technology, proteomic differences were identified between melanoma and benign melanocytic nevi classified with 93% accuracy [24], as well as between Spitz nevi and melanomas showing Spitzoid features, discriminating these tissues with 97% sensitivity and 90% specificity [25]. IMS was found to be more accurate than histopathology in classifying diagnostically challenging atypical Spitzoid neoplasms [26] whose histopathological appearance and the interobserver reproducibility of pathologists for their diagnosis are generally poor. Similarly, Al-Rohil et.al. provided evidence that IMS-based proteomics results in the diagnosis of unambiguous nevi and melanoma were highly concordant (97.8% accuracy) with those obtained by careful histopathologic evaluation from a panel of expert dermatopathologists [27]. An interesting case report from Alomari et.al described how IMS was very helpful in diagnosing a benign nevus in a newborn baby whose mother had stage IV melanoma [28]. IMS is not only applicable for the analysis of proteins/peptides in melanoma tissues but also for measuring and mapping small molecule metabolites in specific sites in the tumor compartments [29]. This finding could help in the characterization of melanoma phenotypes. IMS has also been employed to identify vemurafenib drug distribution in *BRAF* wildtype and *BRAF* V600E mutated tissues following the application of the drug solution onto tissue sections under in vitro conditions. This study demonstrated that drug compounds colocalized with melanoma cells with a higher signal intensity in the *BRAF* mutated tissue than in the wildtype [30].

In our study, we used a proteomic approach-based IMS technology to investigate melanoma tissues carrying *BRAF* and *NRAS* mutations. We investigated the performance of several tissue classifiers, with two types of cross-validation in order to identify proteomic differences characterizing *BRAF* and *NRAS* mutated melanoma.

## 2. Results

**Figure 1:**
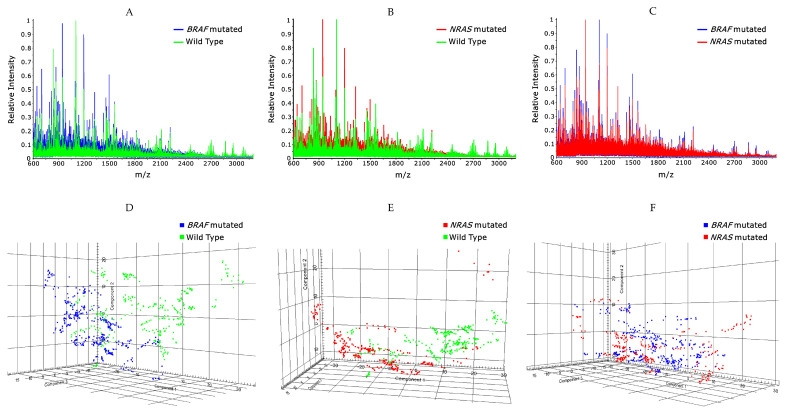
Comparison peptide profile between the average spectrum from *BRAF* (v-raf murine sarcoma viral oncogene homolog B1) mutated (blue) and *BRAF* wildtype (green) melanoma (**A**), *NRAS* (neuroblastoma RAS viral oncogene homolog) mutated (red) and *NRAS* wildtype (green) (**B**), and *BRAF* (blue) and *NRAS* (red) mutated melanoma (**C**). *BRAF* and *NRAS* mutated spectra show different profiles when individually compared to the WT tissues (**A**,**B**), while their profiles show similarity when compared with each other (**C**). Wildtype spectra include both *BRAF* and *NRAS* wildtype patients. Three-dimensional plot of the first three components, resulting from the principal component analysis (PCA), showing separation between *BRAF* mutated (blue) and *BRAF* wildtype (green) (**D**), *NRAS* mutated (red) and *NRAS* wildtype (green) (**E**) spectra. PCA of *BRAF* (blue) and *NRAS* (red) mutated samples show a subtle separation of the two types of spectra (**F**). Each pixel in the PCA corresponds to a spectrum.

Matrix-assisted laser desorption-ionization (MALDI) imaging mass spectrometry (IMS) revealed molecular signatures associated specifically with *BRAF* (v-raf murine sarcoma viral oncogene homolog B1) or *NRAS* (neuroblastoma RAS viral oncogene homolog) mutated or *BRAF*/*NRAS* wildtype (WT) genotypes. Representative mass spectra peptide profiles of *BRAF* (*n* = 411) and *NRAS* (*n* = 381) mutated vs. *BRAF* (*n* = 335) and *NRAS* (*n* = 373) WT spectra, as well as *BRAF* (*n* = 411) vs. *NRAS* (*n* = 381) mutated spectra, are shown in Figure 1A–C. The in situ proteome expression of all samples was analyzed by unsupervised principal component analysis (PCA), resulting in the segregation of spectra. Figure 1D–F shows the 3D scores plot of the first three components in which *BRAF* mutated and *BRAF* WT spectra can be separated (Figure 1D). The same is shown for the PCA of *NRAS* mutated and *NRAS* WT spectra, where two main clusters, each corresponding to a specific genotype, are well separated (Figure 1E). Conversely, low variance is shown between *BRAF* and *NRAS* mutated data, where no clear separation between the different types of mutation is demonstrated (Figure 1F). 

Classification analysis was performed on spectral features (*m*/*z* features) that were selected by the area under the receiver operating characteristics (AUROC) analysis or by a stepwise forward feature selection (FFS). The performance of each classification model was estimated with leave-one-out cross-validation (LOOCV) (leave-one-spectra-out for models applied to spectra, and leave-one-patient-out cross validation (CV) for models applied to patients), k-fold CV, with k = 4 and k = 10 to discriminate *BRAF* and *NRAS* genotypes, and k = 4 to discriminate different *BRAF* mutations. In leave-one-spectra-out CV, one spectrum is used as a testing dataset and the other spectra as the training dataset. Likewise, for a leave-one-patient-out CV, for each patient, the mean spectra are calculated. Thus, one patient is used to test and the remaining patients are used to train the models. The process was repeated for all spectra and for all patients to achieve combined results. In the k-fold CV, 4- and 10-fold CV were applied to linear discriminant analysis (LDA) and support vector machine (SVM) classification models. Specifically, spectral data (individual spectra or mean spectra) were randomly divided to 4 or 10 equisized groups. Each group was used as the testing dataset at a time and the other 3 (for k = 4) or 9 (for k = 10) groups as the training dataset; then, the process was repeated for all 4 or 10 groups. The combined results of all 4 or 10 folds are reported in Table S1.

### 2.1. Classification of BRAF Mutated/Wildtype

Classification analysis to discriminate *BRAF* mutated from WT patients was evaluated using mean spectra for each individual patient (*BRAF* mutated patients *n* = 23, *BRAF* WT patients *n* = 20) and individual spectra (*BRAF* mutated spectra *n* = 411, *BRAF* WT spectra *n* = 335). In the per-spectra strategy, the best classification results were achieved by using a lower number of *m*/*z* features either by AUROC ≥ 0.8 (*n* = 389) or forward feature selection (FFS) (*n* = 6) with both LDA (accuracy = 73–79% using AUROC ≥ 0.8; accuracy= 92–95% using FFS) and SVM (accuracy = 80–84% using AUROC; accuracy = 92–95% using FFS) models (Table 1 and Table 2). A limitation of this classification is that we used a high number of features (features selected by AUROC > 0.7, *n* = 947) compared to samples (total spectra, *n* = 746) (Table 1), which may lead to overfitting of the data, as the model tries to memorize the noise instead of trying to learn important patterns in the data. To assess a potential overfitting of the data, a classification analysis on the same dataset without cross-validation was performed, and the results were compared with those obtained with cross-validation. In this approach, spectral data were split into a training and testing set, and LDA was used as a classification model (Table S2). As a result, the accuracy of the LDA classification without cross-validation (66%, Table S2) was about as good as the LDA classification with k-fold cross-validation (66–69%, Table 1), which indicated not much overfitting. As expected, classification with k = 10-fold cross-validation improved the prediction accuracy to 69% because the model saw different types of patterns at each training stage and a better representation of all the data.

Likewise, in the per-patients approach, the classification performance increased using a lower number of *m*/*z* features (*n*= 542 based on AUROC; *n*= 3 based on FFS). The best classification result was 94% of accuracy using three features selected by FFS with SVM via both 4-fold and 10-fold cross-validation (Table 1 and Table 2).

### 2.2. Classification of NRAS Mutated/Wildtype

When all individual spectra were used, classification analysis based on 15 features, selected by AUROC ≥ 0.8, showed a better discrimination for both LDA (overall accuracy = 77%) and SVM (overall accuracy = 76%) models, compared to the classification that used features selected by AUROC ≥ 0.7, *n* = 455 (overall accuracy = 60%) (Table 3). Similar results were obtained when the mean spectra of individual patients were used to build classification models. Classification performance increased when using only four features selected by AUROC ≥ 0.84, using either LDA with LOOCV (accuracy = 84%) or SVM with LOOCV and 10-fold CV (accuracy = 82%).

In the forward feature selection approach, the most relevant *m*/*z* features were selected one by one, and all possible classification models were calculated, and the ones with the best accuracies were retained. The process continued until either a specific number of features was selected or until the increase in accuracy fell under the specified threshold. Two approaches were considered when all spectra were used, one including 50 and another one including 27 *m*/*z* features. In the last, both LDA and SVM models showed a better discrimination compared with the classification based on 50 features selected, with SVM reaching the highest accuracy of 81% using LOOCV (Table 4).

When the classification was performed on mean spectra of individual patients, 8 *m*/*z* features were selected as the most relevant features in the FFS approach, and the classification accuracy was higher using the SVM model with LOOCV (accuracy = 79%) when compared to the LDA model (62–64%) (Table 4).

### 2.3. Classification of BRAF Mutated and NRAS Mutated Patients

Next, we compared *BRAF* against *NRAS* mutated MM. *n* = 10 *m*/*z* features in the per-spectra analysis and *n* = 19 *m*/*z* in the per-patient analysis were selected by AUROC ≥ 0.7 (Table 5). Classification accuracy was 62–69% per spectra and 61–67% per patient. Classification performance increased by reducing the number of *m*/*z* features selected by AUROC ≥ 0.73, to *n* = 3 per spectra and *n* = 2 per patient, with a resulting accuracy of 65–70% or 67–71%, respectively. The number of spectral features did not significantly increase by using forward feature selection (FFS), by using spectra (*n* = 18), or using patients (features, *n* = 20). However, enhanced classification performance was achieved with the higher number of features selected by the FFS with SVM models (61–76%) and when spectra were analyzed (Table 6).

Some of the peak classifiers selected by FFS and AUROC are reported in Table 7. The ion peaks at *m*/*z* 768.3, 1026.5, 1252.6, 1300.6, and 1336.6 contributed most to the discrimination of *BRAF* mutated and *BRAF* WT classification. The mass ions at *m*/*z* 715.3, 733.3, 807.3, 826.4, 837.4, 910.4, 982.4, 1082.5, 1091.5, 1129.5, 1222.5, 1259.6, 1262.6, 1512.7, 1678.8, 1783.8 were identified as the major contributors to *NRAS* mutated from *NRAS* WT patients. The peaks corresponding to *m*/*z* 944.4, 1825.9, and 2169 were identified as the main discriminating factors for *BRAF* mutated versus *NRAS* mutated malignant melanoma (MM).

## 3. Discussion

According to gene expression profiling analyses, different malignant melanoma (MM) subgroups can be identified that are not discernible on histology alone. Current treatment decisions are based on clinical and genetical parameters that lead to individualized therapy [33,34]. Nonetheless, a high percentage of patients develop therapy resistance and progressive disease [35]. There is a need to expand our understanding of melanoma biology. While driver mutations have been analyzed and identified in MM in many studies, our understanding of the resulting protein expression changes is incomplete and might lead to a better understanding of predicting therapy response and resistance.

In this regard, the capability of mass spectrometry-based proteomics to reveal the proteomic landscape of disease subgroups has been shown in previous studies [24,26,36]. In the current study, Imaging mass spectrometry (IMS)-based proteomic classification was used to describe proteomic differences between *BRAF* (v-raf murine sarcoma viral oncogene homolog B1) and *NRAS* (neuroblastoma RAS viral oncogene homolog) mutated MM and has expanded the knowledge currently available.

In brief, we identified clusters based on spectral proteomic signatures that distinguished samples with and without *BRAF* and *NRAS* mutations with high accuracy of up to 94% and 84% on the patient level. *BRAF* and *NRAS* mutated MM showed a much more similar proteomic signature, and the maximal accuracy used to discriminate both was 76% using different classification algorithms. It is well known that *BRAF* and *NRAS* play a critical role in the RAF (serine/threonine-specific protein kinases)-MEK (mitogen-activated protein kinase)-ERK (extracellular-related kinase) pathway that leads to stimulation of intracellular processes related to gene expression, cell growth, survival, and differentiation [37,38]. Apparently, the proteomic changes that can be detected by MALDI IMS and that are induced by *BRAF* or *NRAS* mutations are greater compared to WT, as compared between both mutations.

Despite the small number of patients in this pilot study, some *m*/*z* species were found to differentiate *BRAF* and *NRAS* groups and have been described previously in MM [23]. Among them, a number of peaks that have been previously identified as histones were also detected in our study. Histones act as structural elements for DNA and play an important role in DNA transcription and replication [39]. In our studies, we detected lower expression of histones in *NRAS* mutated compared to *BRAF* mutated patients. In this regard, it could be speculated that the higher expression of histones in *BRAF* mutated MM as compared to *BRAF* WT and *NRAS* mutated MM is a proteomic reflection of the more aggressive behavior in these tumors [40]. On the other hand, we found lower histone expression on *NRAS* mutated as compared to *NRAS* WT MM, which would not support this hypothesis, as *NRAS* mutated MM shows an inferior prognosis [41,42].

Besides histones, glyceraldehyde-3-phosphate dehydrogenase (GAPDH) has been identified in a previous mass spectrometry study [31] and was found with a lower expression in *BRAF* mutated when compared to *NRAS* mutated patients. GAPDH is a glycolytic enzyme that in addition to its role in energy metabolism participates in numerous cellular functions, including DNA replication and repair [43]. GAPDH has previously been associated with tumor progression [44]. However, its role in *BRAF* as compared to *NRAS* mutated melanoma needs further investigation.

We identified some peptides as predicted proteins for cytokeratins. Keratins comprise one of the major structural components of the cytoskeleton and have critical roles in the regulation of cell migration and adhesion as well as in the maintenance of cell stiffness in both normal and malignant epithelial cells [45,46]. The detection of some keratins, including cytokeratin-8 or cytokeratin-19 observed in this study, was also reported by Chen et al. by reverse transcription (RT)-PCR analysis in melanoma cell lines and tissues [47].

Peptide peaks predictive for S100 calcium binding proteins (S100 A8, S100 A11) were found to be more expressed in the wildtype tumors. S100A8 proteins can interact with components of the cytoskeleton and may mediate their rearrangements and dynamics. S100 A8 is found to be upregulated by anti-inflammatory mediators [48] and by oxidative stress [49], indicating a protective function. Thus, this may explain the higher expression of S100A8 in wildtype than in mutated tumors. Mutations in mitogen-activated protein kinase (MAPK) pathway genes are known to be associated with poor prognosis [50]. The S-100 protein is considered a characteristic immunohistochemical marker for all melanocytic lesions [51,52]. However, the antibody is strongly associated with S100B and is less specific to other S100 proteins [53]. In an IMS analysis, different S100 proteins can be detected from a single tissue to generate multiple signatures that may improve diagnostics, thus reducing the influence of less melanoma-specific S100 family members.

The present study was limited regarding the number of patients included because it was designed as a proof of principle that IMS-based proteomics is suitable to detect proteomic differences in melanoma with different mutational statuses. Thus, studies with larger sample size and further validation are needed to draw definite conclusions. Despite the low number of samples, we demonstrated proteomic differences in MM with a specific mutational background and provided a framework to explore these changes among MM subtypes that may lead to the development of novel diagnostic, prognostic, or predictive markers.

## 4. Materials and Methods

### 4.1. Sample Collection

The sample cohort consisted of archival formalin-fixed paraffin-embedded (FFPE) tissues from 67 patients diagnosed with malignant melanoma (MM) in which mutation analysis for *BRAF* (v-raf murine sarcoma viral oncogene homolog B1) and *NRAS* (neuroblastoma RAS viral oncogene homolog) had previously been performed for clinical purposes. Specimens were divided into three groups based on the mutational status: *BRAF* mutated, *NRAS* mutated, and *BRAF*/*NRAS* WT. Specifically, 23 patients had *BRAF* mutation, 22 patients had *NRAS* mutation, and 22 patients were *BRAF* and *NRAS* WT. Some clinical information of the patient is reported in Table S3.

### 4.2. Reagents and Equipment

All solvents including xylene, isopropanol, ethanol, acetonitrile, and trifluoroacetic acid were purchased from Fisher Scientific, Schwerte, Germany. Indium-tin-oxide-coated (ITO) glass slides, alpha-cyano-4-hydroxycinnamic acid (HCCA) and peptide calibration standard II were obtained from Bruker Daltonik GmbH, Bremen, Germany. Ammonium bicarbonate, glycerol, poly-l-lysine solution and tris buffer were acquired from Sigma Aldrich Chemie, Taufkirchen, Germany. Trypsin was obtained from Promega, Mannheim, Germany, while potassium sulphate was purchased from Carl Roth Karlsruhe, Germany. For the reagent deposition on tissues, an automatic sprayer devise TM-Sprayer, from HTX Technologies, Chapel Hill, NC, USA, was used. High-resolution scanning of the stained slides was performed with a scanner from 3DHISTECH Ltd., Budapest, Hungary, and an Aperio AT2 slide scanner, Leica Biosystems, Wetzlar, Germany. Mass spectra were acquired using a MALDI rapifleX Tissue-typer mass spectrometer from Bruker Daltonik.

### 4.3. Sample Preparation

FFPE tissue sections were cut (3 µm tick) and mounted onto indium-tin-oxide-coated (ITO) glass slides previously coated with 20 µL of poly-l-lysine solution 0.1% (*w*/*v*) in water and dried at 37 °C overnight. Each ITO slide included more than a tissue section. The total samples set spanned 40 ITO slides. Slides were heated at 80 °C for 15 min, then dewaxed in 100% xylene (2 × 5 min) and rehydrated first in 100% isopropanol (5 min), then in ethanol series (100%, 95%, 70%, 50%), washed in each solution for 5 min, and finally in water for 5 s. Slides were then immersed in 10 mM tris buffer pH = 9 and antigen retrieved at 110 °C for 20 min using a decloaking chamber (ZITOMED Systems GmbH BioCare Medical, Berlin, Germany). Trypsin solution (0.025 μg μL^−1^ in 20 mM ammonium bicarbonate, 0.01% glycerol) was sprayed on tissue using the TM-Sprayer in 8 layers with crisscross pattern and 2 mm track spacing, at temperature = 30 °C, 750 mm/min velocity, 0.03 mL min^−1^ flow rate. On-tissue digestion occurred at 50 °C for 2 h in a chamber prepared with saturated potassium sulfate solution to keep 96% humidity. MALDI matrix solution was prepared with 10 mg mL^−1^ a-Cyano-4-hydroxycinnamic acid (HCCA) (dissolved in 70% acetonitrile, 1% trifluoroacetic acid) and deposited on tissues in 4 layers using the same TM sprayer devise with parameters of temperature = 75 °C, 1200 mm/min velocity, 0.120 mL min^−1^ flow rate.

### 4.4. Mass Spectrometry Analysis

MALDI mass spectra were automatically acquired on a rapifleX MALDI Tissue-typer mass spectrometer (Bruker Daltonik) equipped with a Smartbeam 3D laser and operated in positive ion reflector mode, controlled by the Flex Control 4.0 software package. Then, 500 laser shots per spectrum were acquired at a 10 kHz frequency in the range of *m*/*z* 600–3200 with M5 Smartbeam Parameter at 50 µm × 50 µm scan range. External calibration was performed before each new sample in Cubic Enhance mode with nine standard peptides including Bradykinin Fragment 1–7, Angiotensin II, Angiotensin I, Substance P, Bombesin, Renin Substrate, Adrenocorticotropin ACTH clip 1–17, Adrenocorticotropin ACTH clip 18–39, Somatostatin 28 (Bruker Daltonik). Mass spectra were carried out from representative tumor areas previously annotated by a pathologist on a histological serial section. Around 1000–6000 mass spectra, depending on the size of the annotated area, were obtained from each tumor tissue. Spectra processing included TopHat baseline subtraction, which was performed during data acquisition. TopHat algorithm constructs the baseline by means of the morphological TopHat operator, which is defined as the difference between the original spectrum and its morphological opening [54]. Total ion count (TIC) normalization was performed by SCiLS lab software, version 2022b (Bruker Daltonik), and mass shift analysis and alignment was made using R version 4.0.3 (https://www.r-project.org, accessed on 7 September 2021) [55].

### 4.5. Classification Analysis

**Figure 2:**
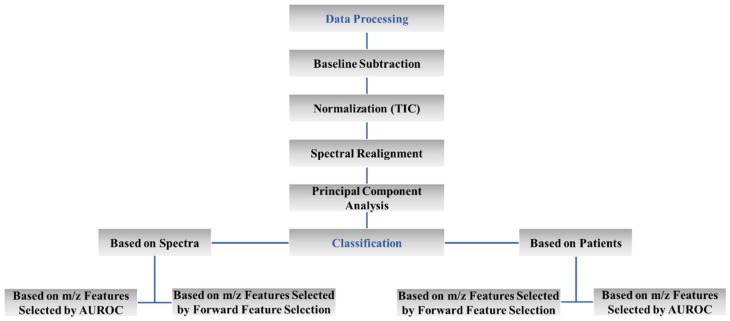
Summary of the data analysis workflow.

Following the MALDI IMS analysis, the same tissue sections were stained with hematoxylin and eosin (HE) and inspected by a pathologist that annotated tumor areas. Histology-annotated images of the tissue sections were merged to the images of the corresponding mass spectrometry data, allowing for extraction of spectral data from areas where more cancer cells were found. Some samples had small tumor areas (~1.5 mm^2^) from which it was possible to recover only a small number of spectra representing tumor (17 spectra). Thus, to perform a consistent data analysis, and based on those samples that had a low number of tumor spectra, it was decided to select around 17 spectra from each tumor region of *BRAF* and *NRAS* mutated and *BRAF* and *NRAS* WT tissue samples. Those selected spectra were used for principal component analysis (PCA), supported by SCiLSLab software, and exported to R version 3.5.3 (https://www.r-project.org, accessed on 7 September 2021) [55] for further classification analysis. Two algorithms including linear discriminant analysis (LDA) and support vector machine (SVM) were applied to classify the three groups of tumors. Classification models are optimized with two internal cross-validation (CV) methods, leave-one-out cross-validation (LOOCV) and k-fold CV. Classification analysis was performed using either individual spectra or the mean spectra calculated over the individual patients. Spectral features (*m*/*z* features) that contributed to the prediction were selected by the area under the receiver operating characteristics (AUROC) analysis or by a stepwise forward feature selection (FFS). In the latter, the model was added in *m*/*z* features one by one so that all possible classification models were calculated and the ones with the best accuracies were retained. The process continued until a specific number of *m*/*z* features was selected, as the model accuracy no longer improved by adding more features. Figure 2 summarizes the data analysis workflow.

## Figures and Tables

**Table 1:** Classification analysis to discriminate *BRAF* (v-raf murine sarcoma viral oncogene homolog B1) mutated (*BRAF* MUT) from *BRAF* wildtype (*BRAF* WT) samples based on *m*/*z* features selected by area under the receiver operating characteristics (AUROC) analysis with AUROC ≥ 0.7, AUROC ≥ 0.8, AUROC ≥ 0.84. Each classification model was performed based on individual spectra and individual patients. Two different classification models were used: linear discriminant analysis (LDA), and support vector machine (SVM). For each model, two cross-validation methods were calculated: leave-one-out cross-validation (LOOCV) and k-fold (k = 4, k = 10) cross-validation.

Samples Used for Classification	Classification Model	AUROC	Features Selected (*n*)	Cross-Validation	Accuracy (%)
Total Spectra, *n* = 746 *BRAF* WT, *n* = 335 *BRAF* MUT, *n* = 411	LDA	≥0.7	947	LOOCV	74
				k-fold (k = 4)	66
				k-fold (k = 10)	69
		≥0.8	389	LOOCV	79
				k-fold (k = 4)	76
				k-fold (k = 10)	73
	SVM	≥0.7	947	LOOCV	80
				k-fold (k = 4)	80
				k-fold (k = 10)	77
		≥0.8	389	LOOCV	84
				k-fold (k = 4)	80
				k-fold (k = 10)	82
Total Patients, *n* = 45 *BRAF* WT, *n* = 22 *BRAF* MUT, *n* = 23	LDA	≥0.7	1057	LOOCV	81
				k-fold (k = 4)	75
				k-fold (k = 10)	81
		≥0.84	542	LOOCV	84
				k-fold (k = 4)	82
				k-fold (k = 10)	82
	SVM	≥0.7	1057	LOOCV	81
				k-fold (k = 4)	78
				k-fold (k = 10)	81
		≥0.84	542	LOOCV	86
				k-fold (k = 4)	84
				k-fold (k = 10)	86

**Table 2:** Classification analysis to discriminate *BRAF* (v-raf murine sarcoma viral oncogene homolog B1) mutated (*BRAF* MUT) from *BRAF* wildtype (*BRAF* WT) samples based on *m*/*z* features selected by forward feature selection (FFS). Each classification model was performed based on individual spectra (*n* = 30 and *n* = 6 features selected) and individual patients (*n* = 30, and *n* = 3 features selected). Two different classification models were used: linear discriminant analysis (LDA), and support vector machine (SVM). For each model two cross-validation methods were calculated: leave-one-out cross-validation (LOOCV) and k-fold (k = 4, k = 10) cross-validation.

Samples Used for Classification	Classification Model	Features Selected ( *n*)	Cross-Validation	Accuracy (%)
Total Spectra, *n* = 746 *BRAF* WT, *n* = 335 *BRAF* MUT, *n* = 411	LDA	30	LOOCV	82
			k-fold (k = 4)	77
			k-fold (k = 10)	83
		6	LOOCV	93
			k-fold (k = 4)	92
			k-fold (k = 10)	95
	SVM	30	LOOCV	80
			k-fold (k = 4)	80
			k-fold (k = 10)	81
		6	LOOCV	95
			k-fold (k = 4)	92
			k-fold (k = 10)	94
Total Patients, *n* = 45 *BRAF* WT, *n* = 22 *BRAF* MUT, *n* = 23	LDA	30	LOOCV	74
			k-fold (k = 4)	61
			k-fold (k = 10)	69
		3	LOOCV	93
			k-fold (k = 4)	91
			k-fold (k = 10)	93
	SVM	30	LOOCV	84
			k-fold (k = 4)	74
			k-fold (k = 10)	81
		3	LOOCV	93
			k-fold (k = 4)	94
			k-fold (k = 10)	94

**Table 3:** Classification analysis to discriminate *NRAS* (neuroblastoma RAS viral oncogene homolog) mutated (*NRAS* MUT) from *NRAS* wildtype (*NRAS* WT) samples based on *m*/*z* features selected by area under the receiver operating characteristics (AUROC) analysis with AUROC ≥ 0.7 and AUROC ≥ 0.8. Each classification model classified individual spectra and individual patients. Two different classification models were used: linear discriminant analysis (LDA), and support vector machine (SVM). For each model two cross-validation methods were calculated: leave-one-out cross-validation (LOOCV) and k-fold (k = 4, k = 10) cross-validation.

Samples Used for Classification	Classification Model	AUROC	Features Selected ( *n*)	Cross-Validation	Accuracy (%)
Total Spectra, *n* = 754 *NRAS* WT, *n* = 373 *NRAS* MUT, *n* = 381	LDA	≥0.7	455	LOOCV	64
				k-fold (k = 4)	52
				k-fold (k = 10)	61
		≥0.8	15	LOOCV	76
				k-fold (k = 4)	76
				k-fold (k = 10)	77
	SVM	≥0.7	455	LOOCV	65
				k-fold (k = 4)	68
				k-fold (k = 10)	63
		≥0.8	15	LOOCV	76
				k-fold (k = 4)	76
				k-fold (k = 10)	76
Total Patients, *n* = 44 *NRAS* WT, *n* = 22 *NRAS* MUT, *n* = 22	LDA	≥0.8	28	LOOCV	57
				k-fold (k = 4)	56
				k-fold (k = 10)	58
		≥0.84	4	LOOCV	84
				k-fold (k = 4)	77
				k-fold (k = 10)	77
	SVM	≥0.8	28	LOOCV	64
				k-fold (k = 4)	66
				k-fold (k = 10)	65
		≥0.84	4	LOOCV	82
				k-fold (k = 4)	81
				k-fold (k = 10)	82

**Table 4:** Classification analysis to discriminate *NRAS* (neuroblastoma RAS viral oncogene homolog) mutated (*NRAS* MUT) from *NRAS* wildtype (*NRAS* WT) samples based on *m*/*z* features selected by forward feature selection (FFS). Each classification model classified individual spectra and individual patients. Two different classification models are used: linear discriminant analysis (LDA) and support vector machine (SVM). For each model, two cross-validation methods were calculated: leave-one-out cross-validation (LOOCV) and k-fold (k = 4, k = 10) cross-validation.

Samples Used for Classification	Classification Model	Features Selected ( *n*)	Cross-Validation	Accuracy (%)
Total Spectra, *n* = 754 *NRAS* WT, *n* = 373 *NRAS* MUT, *n* = 381	LDA	50	LOOCV	71
			k-fold (k = 4)	75
			k-fold (k = 10)	65
		27	LOOCV	78
			k-fold (k = 4)	75
			k-fold (k = 10)	79
	SVM	50	LOOCV	77
			k-fold (k = 4)	72
			k-fold (k = 10)	76
		27	LOOCV	81
			k-fold (k = 4)	79
			k-fold (k = 10)	79
Total Patients, *n* = 44 *NRAS* WT, *n* = 22 *NRAS* MUT, *n* = 22	LDA	8	LOOCV	64
			k-fold (k = 4)	62
			k-fold (k = 10)	63
	SVM	8	LOOCV	79
			k-fold (k = 4)	68
			k-fold (k = 10)	72

**Table 5:** Classification analysis to discriminate *BRAF* (v-raf murine sarcoma viral oncogene homolog B1) (*BRAF* MUT) from *NRAS* (neuroblastoma RAS viral oncogene homolog) (*NRAS* MUT) mutated samples based on *m*/*z* features selected by area under the receiver operating characteristics (AUROC) analysis with AUROC ≥ 0.7 and AUROC ≥ 0.73. Each classification model classified individual spectra and individual patients. Two different classification models were used: linear discriminant analysis (LDA) and support vector machine (SVM). For each model, two cross-validation methods were calculated: leave-one-out cross-validation (LOOCV) and k-fold (k = 4, k = 10) cross-validation.

Samples Used for Classification	Classification Model	AUROC	Features Selected ( *n*)	Cross-Validation	Accuracy (%)
Total Spectra, *n* = 792 *BRAF* MUT, *n* = 411 *NRAS* MUT, *n* = 381	LDA	≥0.7	10	LOOCV	65
				k-fold (k = 4)	62
				k-fold (k = 10)	66
		≥0.73	3	LOOCV	67
				k-fold (k = 4)	68
				k-fold (k = 10)	66
	SVM	≥0.7	10	LOOCV	69
				k-fold (k = 4)	67
				k-fold (k = 10)	67
		≥0.73	3	LOOCV	70
				k-fold (k = 4)	65
				k-fold (k = 10)	70
Total Patients, *n* = 45 *BRAF* MUT, *n* = 23 *NRAS* MUT, *n* = 22	LDA	≥0.7	19	LOOCV	67
				k-fold (k = 4)	64
				k-fold (k = 10)	61
		≥0.73	2	LOOCV	71
				k-fold (k = 4)	67
				k-fold (k = 10)	69
	SVM	≥0.7	19	LOOCV	67
				k-fold (k = 4)	64
				k-fold (k = 10)	61
		≥0.73	2	LOOCV	67
				k-fold (k = 4)	67
				k-fold (k = 10)	67

**Table 6:** Classification analysis to discriminate *BRAF* (v-raf murine sarcoma viral oncogene homolog B1) (*BRAF* MUT) from *NRAS* (neuroblastoma RAS viral oncogene homolog) (*NRAS* MUT) mutated samples based on *m*/*z* features selected by forward feature selection (FFS). Each classification model classified individual spectra and individual patients. Two different classification models were used: linear discriminant analysis (LDA) and support vector machine (SVM). For each model, two cross-validation methods were calculated: leave-one-out cross-validation (LOOCV) and k-fold (k = 4, k = 10) cross-validation.

Samples Used for Classification	Classification Model	Features Selected ( *n*)	Cross-Validation	Accuracy (%)
Total Spectra, *n* = 792 *BRAF* MUT, *n* = 411 *NRAS* MUT, *n* = 381	LDA	18	LOOCV	58
			k-fold (k = 4)	61
			k-fold (k = 10)	58
		10	LOOCV	70
			k-fold (k = 4)	67
			k-fold (k = 10)	63
	SVM	18	LOOCV	76
			k-fold (k = 4)	61
			k-fold (k = 10)	72
		10	LOOCV	71
			k-fold (k = 4)	68
			k-fold (k = 10)	71
Total Patients, *n* = 45 *BRAF* MUT, *n* = 23 *NRAS* MUT, *n* = 22	LDA	19	LOOCV	42
			k-fold (k = 4)	47
			k-fold (k = 10)	44
		2	LOOCV	71
			k-fold (k = 4)	71
			k-fold (k = 10)	67
	SVM	19	LOOCV	53
			k-fold (k = 4)	56
			k-fold (k = 10)	52
		2	LOOCV	69
			k-fold (k = 4)	65
			k-fold (k = 10)	69

**Table 7:** List of the major *m*/*z* peak discriminators selected by forward feature selection (FFS). Tentative identification of proteins between *NRAS* (neuroblastoma RAS viral oncogene homolog) mutated and *NRAS* WT, between *BRAF* (v-raf murine sarcoma viral oncogene homolog B1) mutated and *BRAF* WT, and between *BRAF* mutated and *NRAS* mutated are reported based on tandem mass spectrometry (MS/MS) identifications from the literature.

Classification Comparison	*m*/*z*	Predictive Protein	FSS Accuracy	AUROC	AVG Intensity *BRAF* Mutated	AVG Intensity *NRAS* Mutated	AVG Intensity Wildtype	Log_2_-Fold Change	Ref.
BRAF MUT/WT	768.3	Histone H3.Y	0.98	0.8	3.52		2.31	0.61	[23]
	1026.5	Histone H3.Y	0.89	0.92	5.63		3.18	0.82	[23]
	1252.6	Glyceraldehyde-3-phosphate dehydrogenase	0.97	0.81	3.91		2.88	0.44	[31]
	1300.6	Histone H3.Y	0.9	0.85	4.16		2.65	0.65	[23]
	1336.6	Histone H3.3C	0.95	0.75	2.64		2.09	0.33	[23]
*NRAS* MUT/WT	715.3	Histone H3.3C	0.88	0.92		7.38	15.47	1.067	[23]
	733.3	Histone H3.X	0.93	0.92		2.99	9.57	1.67	[23]
	807.3	Cytochrome c	0.96	0.94		5.32	13.25	1.31	[23]
	826.4	Histone H3.Y	0.93	0.91		2.55	6.8	1.4	[23]
	837.4	Defensin, alpha 6	0.94	0.83		35.48	25.22	−0.49	[23]
	910.4	Histone H3.X	0.92	0.96		4.39	24.18	2.45	[23]
	982.4	Protein S100-A8 (Calgranulin-A)	0.88	0.93		6.64	24.29	1.87	[23]
	1082.5	Keratin, type I cytoskeletal 19	0.91	0.9		4.97	14.93	1.58	[32]
	1091.5	Histone H3.X	0.92	0.87		3.78	12.2	1.69	[23]
	1129.5	Keratin, type II cytoskeletal 8 or Alpha-enolase	0.97	0.76		6.55	7.74	0.24	[32]
	1222.6	Keratin, type 1 cytoskeletal 19 or Keratin, type 1 cytoskeletal 17	0.91	0.85		5.44	9.3	0.77	[32]
	1259.6	S100-A11 (Calgizzarin)	0.93	0.86		4.12	5.44	0.39	[23]
	1262.6	Thymosin beta-4	0.97	0.79		5.81	7.46	0.36	[23]
	1512.7	Thymosin beta-4	0.9	0.73		5.15	13.16	1.35	[23]
	1678.8	Histone H3.X	0.86	0.7		3.94	4.35	0.14	[23]
	1783.8	Defensin, alpha 3/Heat shock protein beta-1 (HspB1)	0.93	0.74		3.63	5.86	0.69	[23]
*BRAF* MUT/*NRAS* MUT	944.4	Histone H3.3C	0.76	0.94	52.37	37.35		0.48	[23]
	1825.9	Glyceraldehyde-3-phosphate dehydrogenase	0.76	0.7	4.67	6.45		−0.46	[31]
	2169	Glyceraldehyde-3-phosphate dehydrogenase	0.74	0.88	2.91	3.7		−0.34	[31]

## Data Availability

The data presented in this study are available on reasonable request from the corresponding author.

## Supplementary Materials

The following supporting information can be downloaded at: https://www.mdpi.com/article/10.3390/ijms24065110/s1.
