# Supplementary material for: Imaging Mass Spectrometry for the Classification of Melanoma Based on BRAF/NRAS Mutational Status
Source: Int J Mol Sci. 2023 Mar 7;24(6):5110. doi: 10.3390/ijms24065110 (PMC10049262; doi:10.3390/ijms24065110)
Supplement: Supplementary file 1 [file ijms-24-05110-s001.zip › ijms-2164622-supplementary.pdf]

Imaging Mass spectrometry-based proteomics for analysis of BRAF/NRAS gene status in malignant melanoma

Authors List

Rita Casadonte, Mark Kriegsmann, Katharina Kriegsmann, Helene Streit, Rolf Rüdiger Melíß, Cornelia S.L. Müller and Joerg Kriegsman

Table S1: classification results including all cross-validation methods

Classification of NRAS mutated (NRAS MUT) from NRAS wild type (NRAS WT)

Classification of individual spectra (*n* =754) using Linear Discriminant Analysis (LDA)

Features selected by AUROC ROC ≥ 0.7 (*n* =445)

LOOCV

	NRAS WT	NRAS MUT	**Accuracy**
NRAS WT	235	138	**0.637931**
NRAS MUT	135	246	

kfold (k=10)

1st)	NRAS WT	NRAS MUT	Accuracy	2nd)	NRAS WT	NRAS MUT	Accuracy	3rd)	NRAS WT	NRAS MUT	Accuracy	**Accuracy AVG**
NRAS WT	218	155	0.6472149	NRAS WT	200	173	0.5265252	NRAS WT	275	98	0.6538462	**0.609195433**
NRAS MUT	111	270		NRAS MUT	184	197		NRAS MUT	163	218		

kfold (k=4)

1st)	NRAS WT	NRAS MUT	Accuracy	2nd)	NRAS WT	NRAS MUT	Accuracy	3rd)	NRAS WT	NRAS MUT	Accuracy	**Accuracy AVG**
NRAS WT	211	162	0.5517241	NRAS WT	214	159	0.5676393	NRAS WT	180	193	0.4469496	**0.522104333**
NRAS MUT	176	205		NRAS MUT	167	214		NRAS MUT	224	157		

Classification of individual spectra (*n* =754) using Support Vector Machine (SVM)

Features selected by AUROC ROC ≥ 0.7 (*n* =445)

LOOCV

	NRAS WT	NRAS MUT	**Accuracy**
NRAS WT	246	127	**0.6472149**
NRAS MUT	135	242	

kfold (k=10)

1st)	NRAS WT	NRAS MUT	Accuracy	2nd)	NRAS WT	NRAS MUT	Accuracy	3rd)	NRAS WT	NRAS MUT	Accuracy	**Accuracy AVG**
NRAS WT	235	138	0.6206897	NRAS WT	254	119	0.7002653	NRAS WT	242	131	0.5782493	**0.6330681**
NRAS MUT	148	233		NRAS MUT	107	274		NRAS MUT	187	194		

kfold (k=4)

1st)	NRAS WT	NRAS MUT	Accuracy	2nd)	NRAS WT	NRAS MUT	Accuracy	3rd)	NRAS WT	NRAS MUT	Accuracy	**Accuracy AVG**
NRAS WT	258	115	0.6962865	NRAS WT	238	135	0.6856764	NRAS WT	240	133	0.6498674	**0.677276767**
NRAS MUT	114	267		NRAS MUT	102	279		NRAS MUT	131	250		

Classification of individual spectra (*n* =754) using Linear Discriminant Analysis (LDA)

Features selected by AUROC ROC ≥ 0.8 (*n* =15)

LOOCV

	NRAS WT	NRAS MUT	**Accuracy**
NRAS WT	267	106	**0.765252**
NRAS MUT	71	310	

kfold (k=10)

1st)	NRAS WT	NRAS MUT	Accuracy	2nd)	NRAS WT	NRAS MUT	Accuracy	3rd)	NRAS WT	NRAS MUT	Accuracy	**Accuracy AVG**
NRAS WT	267	106	0.7533156	NRAS WT	278	95	0.7732095	NRAS WT	283	90	0.7838196	**0.7701149**
NRAS MUT	80	301		NRAS MUT	76	305		NRAS MUT	73	308		

kfold (k=4)

1st)	NRAS WT	NRAS MUT	Accuracy	2nd)	NRAS WT	NRAS MUT	Accuracy	3rd)	NRAS WT	NRAS MUT	Accuracy	**Accuracy AVG**
NRAS WT	248	125	0.7360743	NRAS WT	278	95	0.7440318	NRAS WT	263	110	0.8050398	**0.7617153**
NRAS MUT	74	307		NRAS MUT	98	283		NRAS MUT	37	344		

Classification of individual spectra (*n* =754) using Support Vector Machine (SVM)

Features selected by AUROC ROC ≥ 0.8 (*n* =15)

LOOCV

	NRAS WT	NRAS MUT	**Accuracy**
NRAS WT	273	100	**0.7639257**
NRAS MUT	78	303	

kfold (k=10)

1st)	NRAS WT	NRAS MUT	Accuracy	2nd)	NRAS WT	NRAS MUT	Accuracy	3rd)	NRAS WT	NRAS MUT	Accuracy	**Accuracy AVG**
NRAS WT	272	101	0.7519894	NRAS WT	274	99	0.7864721	NRAS WT	274	99	0.7427056	**0.760389033**
NRAS MUT	86	295		NRAS MUT	62	319		NRAS MUT	95	286		

kfold (k=4)

1st)	NRAS WT	NRAS MUT	Accuracy	2nd)	NRAS WT	NRAS MUT	Accuracy	3rd)	NRAS WT	NRAS MUT	Accuracy	**Accuracy AVG**
NRAS WT	288	85	0.7997347	NRAS WT	263	110	0.7175066	NRAS WT	250	123	0.7533156	**0.7568523**
NRAS MUT	66	315		NRAS MUT	103	278		NRAS MUT	63	318		

Classification of individual spectra (*n* =754) using Linear Discriminant Analysis (LDA)

Features selected by forward feature selection. *N* features selected with early termination =FALSE (*n* =50)

LOOCV

	NRAS WT	NRAS MUT	**Accuracy**
NRAS WT	260	113	**0.7135279**
NRAS MUT	103	278	

kfold (k=10)

1st)	NRAS WT	NRAS MUT	Accuracy	2nd)	NRAS WT	NRAS MUT	Accuracy	3rd)	NRAS WT	NRAS MUT	Accuracy	**Accuracy AVG**
NRAS WT	273	100	0.7374005	NRAS WT	287	86	0.7851459	NRAS WT	258	115	0.7161804	**0.746242267**
NRAS MUT	98	283		NRAS MUT	76	305		NRAS MUT	99	282		

kfold (k=4)

1st)	NRAS WT	NRAS MUT	Accuracy		NRAS WT	NRAS MUT	Accuracy		NRAS WT	NRAS MUT	Accuracy	**Accuracy AVG**
NRAS WT	239	134	0.6525199	NRAS WT	255	118	0.6206897	BRAF WT/NR.	259	114	0.6843501	**0.6525199**
NRAS MUT	128	253		NRAS MUT	168	213		NRAS MUT	124	257		

Classification of individual spectra (*n* =754) using Support Vector Machine (SVM)

Features selected by forward feature selection. *N* features selected with early termination =FALSE (*n* =50)

LOOCV

	NRAS WT	NRAS MUT	**Accuracy**
NRAS WT	277	96	**0.770557**
NRAS MUT	77	304	

kfold (k=10)

1st)	NRAS WT	NRAS MUT	Accuracy	2nd)	NRAS WT	NRAS MUT	Accuracy	3rd)	NRAS WT	NRAS MUT	Accuracy	**Accuracy AVG**
NRAS WT	296	77	0.7188329	NRAS WT	306	67	0.8090186	NRAS WT	284	89	0.7586207	**0.7621574**
NRAS MUT	135	246		NRAS MUT	77	304		NRAS MUT	93	288		

kfold (k=4)

1st)	NRAS WT	NRAS MUT	Accuracy	2nd)	NRAS WT	NRAS MUT	Accuracy	3rd)	NRAS WT	NRAS MUT	Accuracy	**Accuracy AVG**
NRAS WT	304	69	0.7665782	NRAS WT	301	72	0.8156499	NRAS WT	267	106	0.7480106	**0.7241379**
NRAS MUT	107	274		NRAS MUT	67	314		NRAS MUT	102	279		

Classification of individual spectra (*n* =754) using Linear Discriminant Analysis (LDA)

Features selected by forward feature selection. *N* features selected with early termination=FALSE (*n* =27). *N* features >27 did not improve the accuracy

LOOCV

	NRAS WT	NRAS MUT	**Accuracy**
NRAS WT	290	83	**0.7838196**
NRAS MUT	80	301	

kfold (k=10)

1st)	NRAS WT	NRAS MUT	Accuracy	2nd)	NRAS WT	NRAS MUT	Accuracy	3rd)	NRAS WT	NRAS MUT	Accuracy	**Accuracy AVG**
NRAS WT	276	97	0.7665782	NRAS WT	300	73	0.8275862	NRAS WT	302	71	0.7798408	**0.791335067**
NRAS MUT	79	302		NRAS MUT	57	324		NRAS MUT	95	286		

kfold (k=4)

1st)	NRAS WT	NRAS MUT	Accuracy	2nd)	NRAS WT	NRAS MUT	Accuracy	3rd)	NRAS WT	NRAS MUT	Accuracy	**Accuracy AVG**
NRAS WT	295	78	0.7374005	NRAS WT	285	88	0.7267905	NRAS WT	272	101	0.7758621	**0.746684367**
NRAS MUT	120	261		NRAS MUT	118	263		NRAS MUT	68	313		

Classification of individual spectra (*n* =754) using Support Vector Machine (SVM)

Features selected by forward feature selection. *N* features selected with early termination=FALSE (*n* =27). *N* features >27 did not improve the accuracy

LOOCV

	NRAS WT	NRAS MUT	**Accuracy**
NRAS WT	306	67	**0.806366**
NRAS MUT	79	302	

kfold (k=10)

1st)	NRAS WT	NRAS MUT	Accuracy	2nd)	NRAS WT	NRAS MUT	Accuracy	3rd)	NRAS WT	NRAS MUT	Accuracy	**Accuracy AVG**
NRAS WT	304	69	0.801061	NRAS WT	314	59	0.8129973	NRAS WT	291	82	0.770557	**0.794871767**
NRAS MUT	81	300		NRAS MUT	82	299		NRAS MUT	91	290		

kfold (k=4)

1st)	NRAS WT	NRAS MUT	Accuracy	2nd)	NRAS WT	NRAS MUT	Accuracy	3rd)	NRAS WT	NRAS MUT	Accuracy	**Accuracy AVG**
NRAS WT	286	87	0.7838196	NRAS WT	293	80	0.806366	NRAS WT	295	78	0.7917772	**0.7939876**
NRAS MUT	76	305		NRAS MUT	66	315		NRAS MUT	79	302		

Classification based on patients (*n* =44) using Linear Discriminant Analysis (LDA)

Features selected by AUROC ROC ≥ 0.8 (*n* =28)

LOOCV

	NRAS WT	NRAS MUT	**Accuracy**
NRAS WT	11	11	**0.5681818**
NRAS MUT	8	14	

kfold (k=10)

1st)	NRAS WT	NRAS MUT	Accuracy	2nd)	NRAS WT	NRAS MUT	Accuracy	3rd)	NRAS WT	NRAS MUT	Accuracy	**Accuracy AVG**
NRAS WT	7	1015	0.6136364	NRAS WT	13	9	0.5681818	NRAS WT	14	8	0.5681818	**0.583333333**
NRAS MUT				NRAS MUT	10	12		NRAS MUT	11	11		

kfold (k=4)

1st)	NRAS WT	NRAS MUT	Accuracy	2nd)	NRAS WT	NRAS MUT	Accuracy	3rd)	NRAS WT	NRAS MUT	Accuracy	**Accuracy AVG**
NRAS WT	11	11	0.5	NRAS WT	11	11	0.5909091	NRAS WT	13	9	0.5909091	**0.560606067**
NRAS MUT	11	11		NRAS MUT	7	15		NRAS MUT	9	13		

Classification based on patients (*n* =44) using using Support Vector Machine (SVM)

Features selected by AUROC ROC ≥ 0.8 (*n* =28)

LOOCV

	NRAS WT	NRAS MUT	**Accuracy**
NRAS WT	14	8	**0.6363636**
NRAS MUT	8	14	

kfold (k=10)

1st)	NRAS WT	NRAS MUT	Accuracy	2nd)	NRAS WT	NRAS MUT	Accuracy	3rd)	NRAS WT	NRAS MUT	Accuracy	**Accuracy AVG**
NRAS WT	14	6	0.675	NRAS WT	14	8	0.6363636	NRAS WT	13	9	0.6363636	**0.6492424**
NRAS MUT	7	13		NRAS MUT	8	14		NRAS MUT	7	15		

kfold (k=4)

1st)	NRAS WT	NRAS MUT	Accuracy	2nd)	NRAS WT	NRAS MUT	Accuracy	3rd)	NRAS WT	NRAS MUT	Accuracy	**Accuracy AVG**
NRAS WT	11	11	0.5	NRAS WT	11	11	0.5909091	NRAS WT	13	9	0.5909091	**0.560606067**
NRAS MUT	11	11		NRAS MUT	7	15		NRAS MUT	9	13		

Classification based on patients (*n* =44) using Linear Discriminant Analysis (LDA)

Features selected by AUROC ROC ≥ 0.84 (*n* =4)

LOOCV

	NRAS WT	NRAS MUT	**Accuracy**
NRAS WT	17	5	**0.8409091**
NRAS MUT	2	20	

kfold (k=10)

1st)	NRAS WT	NRAS MUT	Accuracy	2nd)	NRAS WT	NRAS MUT	Accuracy	3rd)	NRAS WT	NRAS MUT	Accuracy	**Accuracy AVG**
NRAS WT	16	6	0.7727273	NRAS WT	15	7	0.7727273	NRAS WT	16	6	0.7727273	**0.7727273**
NRAS MUT	4	18		NRAS MUT	3	19		NRAS MUT	4	18		

kfold (k=4)

1st)	NRAS WT	NRAS MUT	Accuracy	2nd)	NRAS WT	NRAS MUT	Accuracy	3rd)	NRAS WT	NRAS MUT	Accuracy	**Accuracy AVG**
NRAS WT	17	5	0.8409091	NRAS WT	16	6	0.7954545	NRAS WT	12	10	0.6818182	**0.772727267**
NRAS MUT	2	20		NRAS MUT	3	19		NRAS MUT	4	18		

Classification based on patients (*n* =44) using using Support Vector Machine (SVM)

Features selected by AUROC ROC ≥ 0.84 (*n* =4)

LOOCV

	NRAS WT	NRAS MUT	**Accuracy**
NRAS WT	16	6	**0.8181818**
NRAS MUT	2	20	

kfold (k=10)

1st)	NRAS WT	NRAS MUT	Accuracy	2nd)	NRAS WT	NRAS MUT	Accuracy	3rd)	NRAS WT	NRAS MUT	Accuracy	**Accuracy AVG**
NRAS WT	16	6	0.8181818	NRAS WT	16	6	0.8181818	NRAS WT	16	6	0.8181818	**0.8181818**
NRAS MUT	2	20		NRAS MUT	2	20		NRAS MUT	2	20		

kfold (k=4)

1st)	NRAS WT	NRAS MUT	Accuracy	2nd)	NRAS WT	NRAS MUT	Accuracy	3rd)	NRAS WT	NRAS MUT	Accuracy	**Accuracy AVG**
NRAS WT	16	6	0.8181818	NRAS WT	15	7	0.7954545	NRAS WT	16	6	0.8181818	**0.810606033**
NRAS MUT	2	20		NRAS MUT	2	20		NRAS MUT	2	20		

Classification based on patients (*n* =44) using Linear Discriminant Analysis (LDA)

Features selected by forward feature selection. *N* features selected with early termination=FALSE (*n* =8)

LOOCV

	NRAS WT	NRAS MUT	**Accuracy**
NRAS WT	13	9	**0.6363636**
NRAS MUT	7	15	

kfold (k=10)

1st)	NRAS WT	NRAS MUT	Accuracy	2nd)	NRAS WT	NRAS MUT	Accuracy	3rd)	NRAS WT	NRAS MUT	Accuracy	**Accuracy AVG**
NRAS WT	13	9	0.6363636	NRAS WT	13	9	0.6136364	NRAS WT	14	8	0.6363636	**0.628787867**
NRAS MUT	7	15		NRAS MUT	8	14		NRAS MUT	8	14		

kfold (k=4)

1st)	NRAS WT	NRAS MUT	Accuracy	2nd)	NRAS WT	NRAS MUT	Accuracy	3rd)	NRAS WT	NRAS MUT	Accuracy	**Accuracy AVG**
NRAS WT	10	12	0.5909091	NRAS WT	14	8	0.6136364	NRAS WT	14	8	0.6590909	**0.621212133**
NRAS MUT	6	16		NRAS MUT	9	13		NRAS MUT	7	15		

Classification based on patients (*n* =44) using using Support Vector Machine (SVM)

Features selected by forward feature selection. *N* features selected with early termination=FALSE (*n* =8)

LOOCV

	NRAS WT	NRAS MUT	**Accuracy**
NRAS WT	17	5	**0.7954545**
NRAS MUT	4	18	

kfold (k=10)

1st)	NRAS WT	NRAS MUT	Accuracy	2nd)	NRAS WT	NRAS MUT	Accuracy	3rd)	NRAS WT	NRAS MUT	Accuracy	**Accuracy AVG**
NRAS WT	16	6	0.7272727	NRAS WT	16	6	0.6818182	NRAS WT	17	5	0.75	**0.719696967**
NRAS MUT	6	16		NRAS MUT	8	14		NRAS MUT	6	16		

kfold (k=4)

1st)	NRAS WT	NRAS MUT	Accuracy	2nd)	NRAS WT	NRAS MUT	Accuracy	3rd)	NRAS WT	NRAS MUT	Accuracy	**Accuracy AVG**
NRAS WT	14	8	0.7272727	NRAS WT	16	6	0.7045455	NRAS WT	13	9	0.6136364	**0.6818182**
NRAS MUT	4	18		NRAS MUT	7	15		NRAS MUT	8	14		

Classification of BRAF mutated (BRAF MUT) from BRAF wild type (BRAF WT)

Classification of individual spectra (*n* =746) using Linear Discriminant Analysis (LDA)

Features selected by AUROC ROC ≥ 0.7 (*n* =947)

LOOCV

	BRAF MUT	WT	**Accuracy**
BRAF MUT	339	72	**0.7399464**
WT	122	213	

kfold (k=10)

1st)	BRAF MUT	WT	Accuracy	2nd)	BRAF MUT	WT	Accuracy	3rd)	BRAF MUT	WT	Accuracy	**Accuracy AVG**
BRAF MUT	350	61	0.6983914	BRAF MUT	321	90	0.7117962	BRAF MUT	296	115	0.6715818	**0.693923133**
WT	164	171		WT	125	210		WT	130	205		

kfold (k=4)

1st)	BRAF MUT	WT	Accuracy	2nd)	BRAF MUT	WT	Accuracy	3rd)	BRAF MUT	WT	Accuracy	**Accuracy AVG**
BRAF MUT	332	79	0.6112601	BRAF MUT	255	156	0.6420912	BRAF MUT	323	88	0.730563	**0.661304767**
WT	211	124		WT	111	224		WT	113	222		

Classification of individual spectra (*n* =746) using using Support Vector Machine (SVM)

Features selected by AUROC ROC ≥ 0.7 (*n* =947)

LOOCV

	BRAF MUT	WT	**Accuracy**
BRAF MUT	342	69	**0.7962466**
WT	83	125	

kfold (k=10)

1st)	BRAF MUT	WT	Accuracy	2nd)	BRAF MUT	WT	Accuracy	3rd)	BRAF MUT	WT	Accuracy	**Accuracy AVG**
BRAF MUT	332	79	0.766756	BRAF MUT	301	72	0.7867232	BRAF MUT	331	80	0.7680965	**0.773858567**
WT	95	240		WT	79	256		WT	93	242		

kfold (k=4)

1st)	BRAF MUT	WT	Accuracy	2nd)	BRAF MUT	WT	Accuracy	3rd)	BRAF MUT	WT	Accuracy	**Accuracy AVG**
BRAF MUT	330	81	0.7600536	BRAF MUT	362	49	0.8418231	BRAF MUT	351	60	0.810992	**0.804289567**
WT	97	242		WT	69	266		WT	81	254		

Classification of individual spectra (*n* =746) using Linear Discriminant Analysis (LDA)

Features selected by AUROC ROC ≥ 0.8 (*n* =389)

LOOCV

	BRAF MUT	WT	**Accuracy**
BRAF MUT	336	75	**0.7882038**
WT	83	252	

kfold (k=10)

1st)	BRAF MUT	WT	Accuracy	2nd)	BRAF MUT	WT	Accuracy	3rd)	BRAF MUT	WT	Accuracy	**Accuracy AVG**
BRAF MUT	327	84	0.727882	BRAF MUT	341	70	0.7493298	BRAF MUT	325	86	0.7184987	**0.7319035**
WT	119	216		WT	117	218		WT	124	211		

kfold (k=4)

1st)	BRAF MUT	WT	Accuracy	2nd)	BRAF MUT	WT	Accuracy	3rd)	BRAF MUT	WT	Accuracy	**Accuracy AVG**
BRAF MUT	364	47	0.7600536	BRAF MUT	319	92	0.7882038	BRAF MUT	332	79	0.7412869	**0.763181433**
WT	132	203		WT	66	269		WT	114	221		

Classification of individual spectra (*n* =746) using using Support Vector Machine (SVM)

Features selected by AUROC ROC ≥ 0.8 (*n* =389)

LOOCV

	BRAF MUT	WT	**Accuracy**
BRAF MUT	371	40	**0.8378016**
WT	81	254	

kfold (k=10)

1st)	BRAF MUT	WT	Accuracy	2nd)	BRAF MUT	WT	Accuracy	3rd)	BRAF MUT	WT	Accuracy	**Accuracy AVG**
BRAF MUT	353	58	0.8243968	BRAF MUT	344	67	0.8042895	BRAF MUT	374	37	0.8458445	**0.8248436**
WT	73	262		WT	79	256		WT	78	257		

kfold (k=4)

1st)	BRAF MUT	WT	Accuracy	2nd)	BRAF MUT	WT	Accuracy	3rd)	BRAF MUT	WT	Accuracy	**Accuracy AVG**
BRAF MUT	344	67	0.7962466	BRAF MUT	357	54	0.8230563	BRAF MUT	335	76	0.7922252	**0.8038427**
WT	85	250		WT	78	257		WT	79	256		

Classification of individual spectra (*n* =746) using Linear Discriminant Analysis (LDA)

Features selected by forward feature selection. *N* features selected with early termination=FALSE (*n* =30)

LOOCV

	BRAF MUT	WT	**Accuracy**
BRAF MUT	362	49	**0.8257373**
WT	81	254	

kfold (k=10)

1st)	BRAF MUT	WT	Accuracy	2nd)	BRAF MUT	WT	Accuracy	3rd)	BRAF MUT	WT	Accuracy	**Accuracy AVG**
BRAF MUT	360	51	0.8538874	BRAF MUT	334	77	0.8002681	BRAF MUT	355	56	0.8270777	**0.827077733**
WT	58	277		WT	72	263		WT	73	262		

kfold (k=4)

1st)	BRAF MUT	WT	Accuracy	2nd)	BRAF MUT	WT	Accuracy	3rd)	BRAF MUT	WT	Accuracy	**Accuracy AVG**
BRAF MUT	352	59	0.7895442	BRAF MUT	304	107	0.730563	BRAF MUT	357	54	0.7922252	**0.770777467**
WT	98	237		WT	94	241		WT	101	234		

Classification of individual spectra (*n* =746) using using Support Vector Machine (SVM)

Features selected by forward feature selection. *N* features selected with early termination=FALSE (*n* =30)

LOOCV

	BRAF MUT	WT	**Accuracy**
BRAF MUT	321	90	**0.7975871**
WT	61	274	

kfold (k=10)

1st)	BRAF MUT	WT	Accuracy	2nd)	BRAF MUT	WT	Accuracy	3rd)	BRAF MUT	WT	Accuracy	**Accuracy AVG**
BRAF MUT	336	75	0.8150134	BRAF MUT	338	73	0.8042895	BRAF MUT	310	65	0.808096	**0.809132967**
WT	63	272		WT	73	262		WT	63	229		

kfold (k=4)

1st)	BRAF MUT	WT	Accuracy	2nd)	BRAF MUT	WT	Accuracy	3rd)	BRAF MUT	WT	Accuracy	**Accuracy AVG**
BRAF MUT	322	89	0.7533512	BRAF MUT	314	97	0.7895442	BRAF MUT	356	55	0.8686327	**0.8038427**
WT	95	240		WT	60	275		WT	43	292		

Classification of individual spectra (*n* =746) using Linear Discriminant Analysis (LDA)

Features selected by forward feature selection. *N* features selected with early termination=FALSE (*n* =6)

LOOCV

	BRAF MUT	WT	**Accuracy**
BRAF MUT	390	21	**0.9356568**
WT	27	308	

kfold (k=10)

1st)	BRAF MUT	WT	Accuracy	2nd)	BRAF MUT	WT	Accuracy	3rd)	BRAF MUT	WT	Accuracy	**Accuracy AVG**
BRAF MUT	394	17	0.9423592	BRAF MUT	398	13	0.9530831	BRAF MUT	401	10	0.9597855	**0.9517426**
WT	26	309		WT	22	313		WT	20	315		

kfold (k=4)

1st)	BRAF MUT	WT	Accuracy	2nd)	BRAF MUT	WT	Accuracy	3rd)	BRAF MUT	WT	Accuracy	**Accuracy AVG**
BRAF MUT	379	32	0.8914209	BRAF MUT	399	12	0.9477212	BRAF MUT	389	22	0.9142091	**0.917783733**
WT	49	286		WT	27	308		WT	42	293		

Classification of individual spectra (*n* =746) using using Support Vector Machine (SVM)

Features selected by forward feature selection. *N* features selected with early termination=FALSE (*n* =6)

LOOCV

	BRAF MUT	WT	**Accuracy**
BRAF MUT	396	15	**0.9463807**
WT	25	310	

kfold (k=10)

1st)	BRAF MUT	WT	Accuracy	2nd)	BRAF MUT	WT	Accuracy	3rd)	BRAF MUT	WT	Accuracy	**Accuracy AVG**
BRAF MUT	398	13	0.9477212	BRAF MUT	397	14	0.9302949	BRAF MUT	400	11	0.9504021	**0.942806067**
WT	26	309		WT	38	297		WT	26	309		

kfold (k=4)

1st)	BRAF MUT	WT	Accuracy	2nd)	BRAF MUT	WT	Accuracy	3rd)	BRAF MUT	WT	Accuracy	**Accuracy AVG**
BRAF MUT	399	12	0.9557641	BRAF MUT	379	32	0.9182306	BRAF MUT	367	44	0.8954424	**0.9231457**
WT	21	314		WT	29	306		WT	34	301		

Classification based on patients (*n* =45) using Linear Discriminant Analysis (LDA)

Features selected by AUROC ROC ≥ 0.7 (*n* =1057)

LOOCV

	BRAF MUT	WT	**Accuracy**
BRAF MUT	20	3	**0.8139535**
WT	6	16	

kfold (k=10)

1st)	BRAF MUT	WT	Accuracy	2nd)	BRAF MUT	WT	Accuracy	3rd)	BRAF MUT	WT	Accuracy	**Accuracy AVG**
BRAF MUT	19	4	0.7906977	BRAF MUT	21	2	0.8372093	BRAF MUT	20	3	0.8139535	**0.8139535**
WT	6	16		WT	6	16		WT	6	16		

kfold (k=4)

1st)	BRAF MUT	WT	Accuracy	2nd)	BRAF MUT	WT	Accuracy	3rd)	BRAF MUT	WT	Accuracy	**Accuracy AVG**
BRAF MUT	19	4	0.7906977	BRAF MUT	17	6	0.744186	BRAF MUT	16	7	0.7209302	**0.751937967**
WT	6	16		WT	6	16		WT	6	16		

Classification based on patients (*n* =45) using using Support Vector Machine (SVM)
Features selected by AUROC ROC ≥ 0.7 (*n* =1057)

LOOCV

	BRAF MUT	WT	**Accuracy**
BRAF MUT	20	3	**0.8139535**
WT	6	16	

kfold (k=10)

1st)	BRAF MUT	WT	Accuracy	2nd)	BRAF MUT	WT	Accuracy	3rd)	BRAF MUT	WT	Accuracy	**Accuracy AVG**
BRAF MUT	21	2	0.8372093	BRAF MUT	19	4	0.7674419	BRAF MUT	20	3	0.7906977	**0.798449633**
WT	6	16		WT	6	14		WT	6	14		

kfold (k=4)

1st)	BRAF MUT	WT	Accuracy	2nd)	BRAF MUT	WT	Accuracy	3rd)	BRAF MUT	WT	Accuracy	**Accuracy AVG**
BRAF MUT	19	4	0.7906977	BRAF MUT	20	3	0.8139535	BRAF MUT	19	4	0.7906977	**0.798449633**
WT	6	16		WT	6	16		WT	6	16		

Classification based on patients (*n* =45) using Linear Discriminant Analysis (LDA)
Features selected by AUROC ROC ≥ 0.8 (*n* =542)

LOOCV

	BRAF MUT	WT	**Accuracy**
BRAF MUT	21	2	**0.8372093**
WT	6	16	

kfold (k=10)

1st)	BRAF MUT	WT	Accuracy	2nd)	BRAF MUT	WT	Accuracy	3rd)	BRAF MUT	WT	Accuracy	**Accuracy AVG**
BRAF MUT	22	1	0.8837209	BRAF MUT	18	5	0.7674419	BRAF MUT	19	4	0.8139535	**0.821705433**
WT	5	17		WT	6	16		WT	5	17		

kfold (k=4)

1st)	BRAF MUT	WT	Accuracy	2nd)	BRAF MUT	WT	Accuracy	3rd)	BRAF MUT	WT	Accuracy	**Accuracy AVG**
BRAF MUT	20	3	0.8372093	BRAF MUT	18	5	0.7906977	BRAF MUT	20	3	0.8372093	**0.821705433**
WT	5	17		WT	5	17		WT	5	17		

Classification based on patients (*n* =45) using using Support Vector Machine (SVM)

Features selected by AUROC ROC ≥ 0.8 (*n* =542)

LOOCV

	BRAF MUT	WT	**Accuracy**
BRAF MUT	21	2	**0.8604651**
WT	5	17	

kfold (k=10)

1st)	BRAF MUT	WT	Accuracy	2nd)	BRAF MUT	WT	Accuracy	3rd)	BRAF MUT	WT	Accuracy	**Accuracy AVG**
BRAF MUT	21	2	0.8604651	BRAF MUT	20	3	0.8372093	BRAF MUT	22	1	0.8837209	**0.8604651**
WT	5	17		WT	5	17		WT	5	17		

kfold (k=4)

1st)	BRAF MUT	WT	Accuracy	2nd)	BRAF MUT	WT	Accuracy	3rd)	BRAF MUT	WT	Accuracy	**Accuracy AVG**
BRAF MUT	20	3	0.8372093	BRAF MUT	21	2	0.8604651	BRAF MUT	19	4	0.8139535	**0.8372093**
WT	5	17		WT	5	17		WT	5	17		

Classification based on patients (*n* =45) using Linear Discriminant Analysis (LDA)

Features selected by forward feature selection. *N* features selected with early termination=FALSE (*n* =30)

LOOCV

	BRAF MUT	WT	**Accuracy**
BRAF MUT	17	6	**0.744186**
WT	6	16	

kfold (k=10)

1st)	BRAF MUT	WT	Accuracy	2nd)	BRAF MUT	WT	Accuracy	3rd)	BRAF MUT	WT	Accuracy	**Accuracy AVG**
BRAF MUT	17	6	0.6976744	BRAF MUT	16	7	0.6744186	BRAF MUT	15	8	0.6976744	**0.689922467**
WT	8	14		WT	8	14		WT	6	16		

kfold (k=4)

1st)	BRAF MUT	WT	Accuracy	2nd)	BRAF MUT	WT	Accuracy	3rd)	BRAF MUT	WT	Accuracy	**Accuracy AVG**
BRAF MUT	13	10	0.6744186	BRAF MUT	11	12	0.5116279	BRAF MUT	16	7	0.6511628	**0.6124031**
WT	5	17		WT	10	12		WT	9	13		

Classification based on patients (*n* =45) using using Support Vector Machine (SVM)

Features selected by forward feature selection. *N* features selected with early termination=FALSE (*n* =30)

LOOCV

	BRAF MUT	WT	**Accuracy**
BRAF MUT	20	3	**0.8372093**
WT	5	17	

kfold (k=10)

1st)	BRAF MUT	WT	Accuracy	2nd)	BRAF MUT	WT	Accuracy	3rd)	BRAF MUT	WT	Accuracy	**Accuracy AVG**
BRAF MUT	20	3	0.8139535	BRAF MUT	20	3	0.8372093	BRAF MUT	19	4	0.7906977	**0.8139535**
WT	6	16		WT	5	17		WT	6	16		

kfold (k=4)

1st)	BRAF MUT	WT	Accuracy	2nd)	BRAF MUT	WT	Accuracy	3rd)	BRAF MUT	WT	Accuracy	**Accuracy AVG**
BRAF MUT	17	6	0.7209302	BRAF MUT	20	3	0.7674419	BRAF MUT	18	5	0.744186	**0.744186033**
WT	7	15		WT	8	14		WT	6	16		

Classification based on patients (*n* =45) using Linear Discriminant Analysis (LDA)

Features selected by forward feature selection. *N* features selected with early termination=FALSE (*n* =3)

LOOCV

	BRAF MUT	WT	**Accuracy**
BRAF MUT	23	0	**0.9302326**
WT	4	18	

kfold (k=10)

1st)	BRAF MUT	WT	Accuracy	2nd)	BRAF MUT	WT	Accuracy	3rd)	BRAF MUT	WT	Accuracy	**Accuracy AVG**
BRAF MUT	23	0	0.952381	BRAF MUT	23	0	0.9302326	BRAF MUT	23	0	0.9069767	**0.929863433**
WT	3	19		WT	4	18		WT	4	16		

kfold (k=4)

1st)	BRAF MUT	WT	Accuracy	2nd)	BRAF MUT	WT	Accuracy	3rd)	BRAF MUT	WT	Accuracy	**Accuracy AVG**
BRAF MUT	23	0	0.9534884	BRAF MUT	23	0	0.9302326	BRAF MUT	20	3	0.8604651	**0.9147287**
WT	3	19		WT	4	18		WT	4	18		

Classification based on patients (*n* =45) using using Support Vector Machine (SVM)

Features selected by forward feature selection. *N* features selected with early termination=FALSE (*n* =3)

LOOCV

	BRAF MUT	WT	**Accuracy**
BRAF MUT	22	1	**0.9302326**
WT	3	19	

kfold (k=10)

1st)	BRAF MUT	WT	Accuracy	2nd)	BRAF MUT	WT	Accuracy	3rd)	BRAF MUT	WT	Accuracy	**Accuracy AVG**
BRAF MUT	22	1	0.9302326	BRAF MUT	23	0	0.9534884	BRAF MUT	22	1	0.9302326	**0.937984533**
WT	3	19		WT	3	19		WT	3	19		

kfold (k=4)

1st)	BRAF MUT	WT	Accuracy	2nd)	BRAF MUT	WT	Accuracy	3rd)	BRAF MUT	WT	Accuracy	**Accuracy AVG**
BRAF MUT	23	0	0.9534884	BRAF MUT	22	1	0.9302326	BRAF MUT	23	0	0.9534884	**0.945736467**
WT	3	19		WT	3	19		WT	3	19		

Table S2. Classification of BRAF mutated (BRAF MUT) from wild type (WT) without cross-validation

Classification of individual spectra (*n* =746) using Linear Discriminant Analysis (LDA)

Features selected by AUROC ROC ≥ 0.7 (*n* =947)

Samples used for classification

	N spectra used in the training set	*N* spectra used in the testing set
BRAF MUT	263	148
WT	197	138

Classification results of the testing dataset

	BRAF MUT	WT	**Accuracy**
BRAF MUT	106	42	**0.6643**
WT	84	54	

Table S3. Clinical information of the patients

Subject ID	Gene Status Melanoma	Mutation Type	Age	Gender
1	BRAF MUT	BRAF V600E	50	M
2	BRAF MUT	BRAF V600E	53	F
3	BRAF MUT	BRAF V600E	54	M
4	BRAF MUT	BRAF V600K	60	M
5	BRAF MUT	BRAF V600E	65	M
6	BRAF MUT	BRAF V600E	66	M
7	BRAF MUT	BRAF V600K	68	M
8	BRAF MUT	BRAF V600E	68	F
9	BRAF MUT	BRAF V600E	69	M
10	BRAF MUT	BRAF V600E	69	M
11	BRAF MUT	BRAF V600K/V600R/V600M	70	F
12	BRAF MUT	BRAF V600E	73	M
13	BRAF MUT	BRAF V600E	76	F
14	BRAF MUT	BRAF V600K	78	M
15	BRAF MUT	BRAF V600R	81	F
16	BRAF MUT	BRAF V600E	81	M
17	BRAF MUT	BRAF V600E	82	F
18	BRAF MUT	BRAF V600K	82	M
19	BRAF MUT	BRAF V600E	83	M
20	BRAF MUT	BRAF V600E	83	F
21	BRAF MUT	BRAF V600K	85	F
22	BRAF MUT	BRAF V600E	87	F
23	BRAF MUT	BRAF V600E	87	M
24	NRAS MUT	NRAS 61 Q>R	41	F
25	NRAS MUT	NRAS 61 Q>R	44	M
26	NRAS MUT	NRAS 61 Q>K	51	F
27	NRAS MUT	NRAS 61 Q>L	53	M
28	NRAS MUT	NRAS 61 Q>R	60	M
29	NRAS MUT	NRAS 61 Q>R	60	M
30	NRAS MUT	NRAS 61 Q>K	67	F
31	NRAS MUT	NRAS 61 Q>R	67	M
32	NRAS MUT	NRAS 61 Q>R	68	F
33	NRAS MUT	NRAS 61 Q>K	71	M
34	NRAS MUT	NRAS 61 Q>R	72	M
35	NRAS MUT	NRAS 61 Q>R	75	F
36	NRAS MUT	NRAS 61 Q>K	79	M
37	NRAS MUT	NRAS 61 Q>R	81	F
38	NRAS MUT	NRAS 61 Q>R	81	M
39	NRAS MUT	NRAS 61 Q>K	82	F
40	NRAS MUT	NRAS 61 Q>R	86	M
41	NRAS MUT	NRAS 61 Q>K	86	M
42	NRAS MUT	NRAS 61 Q>R	86	M
43	NRAS MUT	NRAS 61 Q>R	86	M
44	NRAS MUT	NRAS 61 Q>K	89	F
45	NRAS MUT	NRAS 61 Q>H	93	M
46	BRAF WT/NRAS WT	N/A	82	M
47	BRAF WT/NRAS WT	N/A	68	F
48	BRAF WT/NRAS WT	N/A	59	M
49	BRAF WT/NRAS WT	N/A	45	M
50	BRAF WT/NRAS WT	N/A	49	F
51	BRAF WT/NRAS WT	N/A	65	M
52	BRAF WT/NRAS WT	N/A	87	F

53	BRAF WT/NRAS WT	N/A	87	F
54	BRAF WT/NRAS WT	N/A	57	F
55	BRAF WT/NRAS WT	N/A	83	M
56	BRAF WT/NRAS WT	N/A	81	F
57	BRAF WT/NRAS WT	N/A	79	M
58	BRAF WT/NRAS WT	N/A	55	M
59	BRAF WT/NRAS WT	N/A	74	F
60	BRAF WT/NRAS WT	N/A	84	F
61	BRAF WT/NRAS WT	N/A	63	M
62	BRAF WT/NRAS WT	N/A	83	F
63	BRAF WT/NRAS WT	N/A	92	M
64	BRAF WT/NRAS WT	N/A	55	M
65	BRAF WT/NRAS WT	N/A	55	F
66	BRAF WT/NRAS WT	N/A	68	M
67	BRAF WT/NRAS WT	N/A	36	F
